# Identification of ORM1, vWF, SPARC, and PPBP as immune-related proteins involved in immune thrombocytopenia by quantitative LC-MS/MS

**DOI:** 10.1186/s12014-023-09413-0

**Published:** 2023-06-24

**Authors:** Dong-mei Yin, Dai Yuan, Rui-jie Sun, Hong-zhi Xu, Shou-yong Hun, Xiao-hui Sui, Ning-ning Shan

**Affiliations:** 1 Department of Blood Transfusion, Shandong Provincial Hospital, Shandong University, Jinan, Shandong 250021 China; 2 Department of Blood Transfusion, Shandong Provincial Hospital Affiliated to Shandong First Medical University, Jinan, Shandong 250021 China; 3 Department of Hematology, Shandong Provincial Hospital, Shandong University, Jinan, Shandong 250021 China; 4 Department of Hematology, Shandong Provincial Hospital Affiliated to Shandong First Medical University, 324 Jing Wu Rd, Jinan, 250021 Shandong China; 5 Department of Rheumatology, Clinical Immunology Center, Peking Union Medical College Hospital, Beijing, 100000 China

**Keywords:** Immune thrombocytopenia, LC-MS/MS, TNF-α

## Abstract

**Background:**

Immune thrombocytopenia (ITP) is a common autoimmune disease characterized by loss of immune tolerance to platelet autoantigens leading to excessive destruction and insufficient production of platelets.

**Method:**

Quantitative liquid chromatography tandem mass spectrometry (LC-MS/MS) was performed to detect the differentially expressed proteins in bone marrow samples from active ITP patients and normal controls.

**Result:**

Our bioinformatic analysis identified two upregulated proteins (ORM1 and vWF) and two downregulated proteins (PPBP and SPARC) related to immune function. The four proteins were all found to be related to the tumor necrosis factor (TNF) -α signalling pathway and involved in the pathogenesis of ITP in KEGG pathway analysis.

**Conclusion:**

Bioinformatics analysis identified differentially expressed proteins in bone marrow that are involved in the TNF-α signalling pathway and are related to the activation of immune function in ITP patients. These findings could provide new ideas for research on the loss of immune tolerance in ITP patients.

**Supplementary Information:**

The online version contains supplementary material available at 10.1186/s12014-023-09413-0.

## Introduction

Immune thrombocytopenia (ITP) is an acquired autoimmune disease that is characterized by increased destruction and decreased formation of platelets due to autoimmune dysregulation [1]. The mechanism of ITP mainly involves loss of immune tolerance. Platelet membrane glycoproteins (GPs), especially GPIIb/IIIa and GPIb/IX, are recognized by autoreactive T cells and thus can activate B cells to produce anti-platelet antibodies. These anti-platelet antibodies can then bind to the platelet membrane antigen, leading to elimination of sensitized platelets by the monocyte-macrophage system [2–5]. Dysregulation of T cell activity and abnormal expression of cytokines have been suggested to be involved in immune intolerance in ITP [6, 7].

Megakaryocytes are mature cells that originate from haematopoietic stem cells and produce platelets in bone marrow [8]. Megakaryopoiesis is a complex process that involves the complete differentiation of megakaryocytes progenitors into functional platelets and occurs specifically in bone marrow. Impaired maturation of megakaryocytes has been proven to be one of the mechanisms of ITP [9]. Thus, bone marrow is an important medium for ITP research.

Proteomics has become critical for biological research and is recognized as a key method for novel biomarker discovery and personalized therapy development [10]. Parallel reaction monitoring (PRM) is a kind of targeted quantitative proteomics with better specificity, sensitivity and repeatability which can quantify multiple target proteins in complex samples at the same time. The PRM-based targeted method is an efficient approach for samples that are available in limited amounts due to various reasons [11]. So PRM assay has emerged as an alternative targeted method of quantification. In this study, we used quantitative liquid chromatography tandem mass spectrometry (LC-MS/MS) analysis to identify differentially expressed immune-related proteins in bone marrow. Parallel reaction monitoring (PRM) mass spectrometry was used to quantify the target proteins between ITP and control samples with the aim of identifying a new strategy for ITP research.

## Materials and methods

### Patients and control subjects

The bone marrow was collected from twenty newly diagnosed active ITP patients (12 females and 8 males; age range: 18-70years; median age: 42years) and twenty normal controls (12 females and 8 males; age range, 18–55 years; median age, 46 years) at the Department Hematology of Shandong Provincial Hospital during January 2019 to December 2019. All patients fit the newly published criteria and none of them had been treated with glucocorticoids prior to sampling [12]. The platelet counts were from 1$\times$10^9^/L to 30$\times$10^9^ /L, with a median count of 11$\times$10^9^/L (Table 1). Patients with diabetes, hypertension, cardiovascular diseases, pregnancy, active or chronic infections, or connective tissue diseases such as systemic lupus erythematosus (SLE) were excluded. We determined the number of samples according to the actual number of ITP patients fit the criteria above during January 2019 to December 2019. Patients and controls samples were divided into four groups respectively based the similar age and sex composition to reduce the differences between groups and increase the accuracy of the results. However, there were no statistical difference in the sex, age and platelet counts between patient groups and normal groups after statistical analysis. The samples were collected with heparin anticoagulant tube and then the mononuclear cells were separated with lymphocyte separation solution and stored in – 80℃ freezers for the future test.

**Table 1:** Clinical characteristics of ITP patients

**Group 1**					
**Sex/Age (year)**	F/18	M/27	F/41	M/45	F/65
**Bleeding symptoms**	EC	NONE	PT, GUH	EC, GH	EP, GH
**Platelet counts (× 10** ^**9**^ **/L)**	30	18	17	8	29
**Group 2**					
**Sex/Age (year)**	F/18	M/33	F/48	F/43	M/60
**Bleeding symptoms**	PT	GH	GH	EP, GH	EC, GH
**Platelet counts (× 10** ^**9**^ **/L)**	11	11	12	4	4
**Group3**					
**Sex/Age (year)**	F/19	F/38	F/43	M/55	M/70
**Bleeding symptoms**	PT, EC	PT, GUH	EP, GH	GH	NONE
**Platelet counts (× 10** ^**9**^ **/L)**	9	7	12	3	14
**Group 4**					
**Sex/Age (year)**	M/25	M/39	F/43	F/48	F/69
**Bleeding symptoms**	GH, EP	GH	PT, GH	PT, GH	PT, GUH
**Platelet counts (× 10** ^**9**^ **/L)**	12	14	1	3	8
**Median (min–max)**					
**Age (year)**	42 (18–70)				
**Platelet counts (× 10** ^**9**^ **/L)**	11 (1–30)				

PT = Petechiae, EC = ecchymoses, EP = epistaxis, GUH = genitourinary hemorrhage, GH = gingival hemorrhage

Informed consent was obtained from each participating patient and/or legal guardian. The ethical protocol followed guidelines of the research code and the research ethics committee of Shandong Provincial Hospital Affiliated to Shandong University and Shandong Provincial Hospital Affiliated to Shandong First Medical University.

### Protein extraction and trypsin digestion

Sample was sonicated three times on ice using a high intensity ultrasonic processor in lysis buffer (8 M urea, 1% Protease Inhibitor Cocktail), Then the protein extraction and the trypsin digestion were conducted according to the previous study [13, 14]. Briefly, the samples were centrifuged at 12,000 g for 10 min at 4° C to remove cell debris and the supernatant was collected to a new centrifuge tube. After removing the high abundant protein by following the Pierce™ Top 12 Abundant Protein Depletion Spin Columns kit (Thermo, Waltham, USA), the concentration determination was tested with BCA kit (Beyotime Biotechnology, Shanghai, China) according to the manufacturer’s instructions. The protein solution was reduced with 5 mM dithiothreitol (Sigma-Aldrich, Saint Louis, USA) for 30 min at 56 °C and alkylated with 11 mM iodoacetamide (Sigma-Aldrich, Saint Louis, USA) for 15 min at room temperature in darkness. Next, the urea concentration of the sample was diluted to less than 2 M. The protein solution was digested with trypsin (Promega, Madison, USA) at a trypsin/protein ratio of 1:50 (w/w) overnight and at a trypsin/protein ratio of 1:100 (w/w) for an additional 4 h-digestion.

### HPLC fractionation and LC-MS/MS analysis

The peptides were fractionated by high-pH reverse HPLC with the chromatographic column Agilent 1260 (5 µ m particle size, 4.6 mm inner diameter, 250 mm length) (Agilent, USA). After being dissolved in solvent A [0.1% formic acid (Sigma) in 2% acetonitrile (ThermoFisher), the peptides were separated by EASY-nLC 1000 UPLC system (Thermo, USA). The gradient elution was similar to our previous study [13, 14].

The peptides were subjected to NSI source followed by tandem mass spectrometry (MS/MS) in Q Exactive™ Plus (Thermo, Germany) coupled online to the UPLC. The electrospray voltage applied was 2.4 kV. The m/z scan range was 385 to 1500 for full scan, and intact peptides were detected in the Orbitrap at a resolution of 60,000. Peptides were then selected for MS/MS using NCE setting as 30 and the fragments were detected in the Orbitrap at a resolution of 15,000. A data-dependent procedure (DDA) that alternated between one MS scan followed by 20 MS/MS scans with 15.0s dynamic exclusion. Automatic gain control (AGC) was set at 5E4. Fixed first mass was set as 100 m/z.

### Database searching

The resulting MS/MS data was proceeded with MaxQuant search engine (v.1.5.2.8). Tandem mass spectra were searched against human Uniprot database concatenated with reverse decoy database. Trypsin/P was specified as cleavage enzyme allowing up to 2 missing cleavages. The minimum peptide length was set to 7 amino acid residues and the maximum number of modifications was set to 5. The mass tolerance for precursor ions was set as 20 ppm in First search and 5 ppm in Main search, and the mass tolerance for fragment ions was set as 0.02 Da. Carbamidomethyl on Cys was specified as fixed modification and acetylation modification and oxidation on Met were specified as variable modifications. FDR was adjusted to < 1% and minimum score for modified peptides was set > 40.

## Parallel reaction monitoring (PRM) analysis

The quantitative identification of immune-related proteins was processed using targeted proteome quantification technique based on mass spectrometry-parallel reaction monitoring (PRM). The proteins used for PRM validation were based on the results of quantitative proteomic analysis of bone marrow serum above. The procedure of protein extraction and trypsin digestion was similar to those mentioned above. After being separated by ESAY-NLC 1000 UPLC system (Thermo, USA), the peptides were implanted into NSI ion source for ionization and tandem mass spectrometry analysis by Q Exactive TM plus. The PRM data were processed using Skyline (v.3.6) software. The electrospray voltage applied was 2.0 kV. The m/z scan range was 365 to 970 for full scan, and intact peptides were detected in the Orbitrap at a resolution of 70,000. Peptides were then selected for MS/MS using NCE setting as 27 and the fragments were detected in the Orbitrap at a resolution of 17,500. Automatic gain control (AGC) was set at 3E6 for full MS and 1E5 for MS/MS. The maximum IT was set at 160 ms for full MS and auto for MS/MS. The isolation window for MS/MS was set at 1.6 m/z. The transitions in the PRM list were as followed: precursor charges were set as 2, 3, ion charges were set as 1, 2, ion types were set as b, y. The product ions were set as from ion 3 to last ion, the ion match tolerance was set as 0.02 Da.

### Bioinformatics analysis

Gene Ontology (GO) annotation proteome was derived from the UniProt-GOA database(http://www.ebi.ac.uk/GOA/) [15]. InterProScan, an algorithm software based on protein sequence, predicted the GO function of the protein in the case of certain identified proteins without annotations in Uniprot-GOA and then classified the proteins according to cellular composition, molecular function, or biological process. The protein domain functional description identified in this study were annotated by InterProScan (http://www.ebi.ac.uk/interpro/). We used KEGG pathway database (v.2.0, http://www.genome.jp/kaas-bin/kaas_main) to annotate the protein pathway and then matched into the corresponding pathways in the database by KEGG mapper. Wolfpsort (v.0.2, http://www.genscript.com/psort/wolf_psort.html)was used to annotate the subcellular localization of the protein [16]. For functional enrichment, including the enrichment of GO analysis, protein domain and KEGG pathway, a two-tailed Fisher’s exact test was used to test the enrichment of the differentially expressed protein in the all identified proteins, and the analysis with a P-value < 0.05 was considered significant. The clustering relationship was visualized by using the Heatmap.2 function from the R language ‘gplots’ package (v.2.0.3, https://cran.r-project.org/web/packages/cluster/). All differentially expressed protein database accession or sequence were searched against the STRING database version 10.5 for protein-protein interactions [17]. Only interactions between the proteins belonging to the searched data set were selected, thereby excluding external candidates. STRING defines a metric called “confidence score” to define interaction confidence; we fetched all interactions that had a confidence score ≥ 0.7 (high confidence). Interaction network form STRING was visualized in R package “networkD3”.

## Results

### Overview of protein identification

In this study, altogether 829 proteins were identified based on 5130 unique peptides in human bone marrow mononuclear cells (BMMCs). The confident identification of proteins required an FDR < 0.01, therefore, 613 proteins met the above criteria. To check the acquired MS data, we verified that the mass error was between – 5 and 5 ppm, which meets the requirement of mass accuracy. Most peptides ranged from 7 to 20 amino acids in length, which is consistent with the basic principle of trypsin digestion The cutoff for the identification of proteins or differentially expressed proteins was a P value < 0.05. A greater than 1.5-fold change was termed upregulation, while a fold change of < 1/1.5 was termed downregulation for proteins. Of the differentially expressed proteins between ITP patients and controls, 26 were upregulated and 69 were downregulated including ORM1, vWF, PPBP and SPARC.

### Functional enrichment of immune-related proteins

To understand the identified proteins and quantified proteins in the database searching process, we annotated the proteins in details from gene ontology (GO), protein domain and KEGG pathway. After annotation, we further performed a GO, KEGG pathway, and protein domain enrichment analysis with the intention of assessing whether there was a significant protein enrichment trend in some functional categories. A bubble chart obtained by the two-tailed Fisher’s exact test was employed to show the enrichment of the differentially expressed protein against all identified proteins. A P-value less than 0.05 was considered statistically significant.

**Fig. 1:**
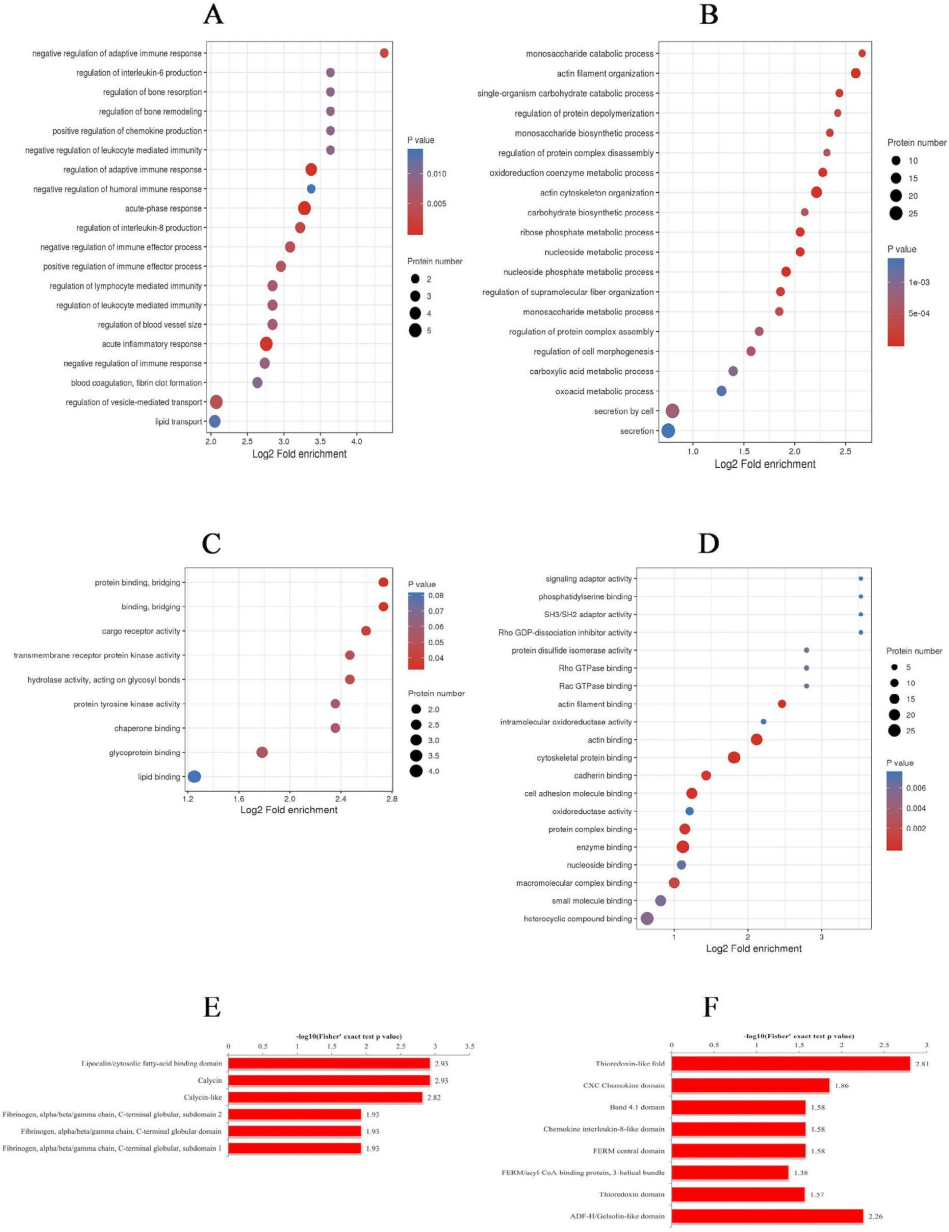
Fisher’s exact test was used for functional classification and to obtain the pathways with significant protein enrichment (P < 0.05) **A**: The biological process enriched in upregulated proteins **B**: Enrichment analysis of the biological processes of the downregulated proteins. **C**: Enrichment analysis of the molecular functions of the upregulated proteins. **D**: Enrichment analysis of the molecular functions of the downregulated proteins **E**: Domain enrichment analysis of the upregulated proteins. **F**: Domain enrichment analysis of the downregulated proteins

The biological processes enrichment of the differentially expressed proteins showed that the upregulated proteins were mainly enriched in the acute-phase response, acute inflammatory response, regulation of interleukin (IL)-6 production and regulation of leukocyte-mediated immunity terms, with which ORM1 was associated, as well as the blood coagulation and fibrin clot formation terms, with which vWF was associated (Fig. 1a). The downregulated proteins were mainly enriched in the regulation of cell morphogenesis term, with which SPARC was associated, and in the secretion by cell term, with which PPBP was associated (Fig. 1b). In the molecular function category, the upregulated proteins were enriched in the binding and protein binding terms, which were related to the immune-related proteins ORM1 and vWF, and in the glycoprotein binding term, which was related to vWF (Fig. 1c). In the downregulated proteins analysis, a certain number of proteins were enriched in the cytoskeletal protein binding and enzyme binding terms; terms related to immunity, protein complex binding and macromolecular complex binding were all associated with SPARC (Fig. 1d).

The KEGG-based functional enrichment analysis showed that the upregulated proteins were enriched in complement and coagulation cascades, in which vWF participates. vWF was also involved in the PI3K-Akt signalling pathway, focal adhesion and ECM-receptor interaction (Supplementary Table S1), which are associated with the TNF-α signalling pathway (Supplementary Fig. 1). While the downregulated proteins were enriched in the carbon metabolism, biosynthesis of amino acids and glycolysis/gluconeogenesis pathways. There were other enriched KEGG functional pathways. For example, the cytokine-cytokine receptor interactions and chemokine signalling pathway which were related to PPBP maybe associated with the immune function in ITP. (Supplementary Table S1).

Furthermore, the domain enrichment of the upregulated proteins was enriched in lipocalin/cytosolic fatty-acid binding domain, calycin domain and calycin-like domain, which were associated with the immune-related protein ORM1 (Fig. 1e). In the downregulated protein analysis, a certain number of proteins were enriched in thioredoxin-like fold. Other CXC chemokine domain and chemokine IL-8-like domain existed in PPBP (Fig. 1f).

### Cluster analysis

Based on the analysis of proteins in the different comparison groups for the GO classification, KEGG pathway and protein domain enrichment, we applied cluster analysis in the comparison groups to assess the correlations between the functions of differentially expressed proteins. All the categories obtained in the enrichment analysis along with their P values were first collated for further hierarchical clustering based on the functional classifications of the differentially expressed proteins. Then, the categories that were enriched in at least one cluster with a P value < 0.05 were filtered. We divided the differentially expressed proteins into four parts according to their fold changes, quartile (Q) 1 through Q4. Specifically, the proteins were divided according to their P/C ratios (Q1, 0 < P/C ratio < 1/2; Q2, 1/2 < P/C ratio < 1/1.5; Q3, 1.5 < P/C ratio < 2; and Q4, P/C ratio > 2), as shown in the figure. The upregulated proteins were classified into Q3 and Q4, while the downregulated proteins were classified into Q1 and Q2.

**Fig. 2:**
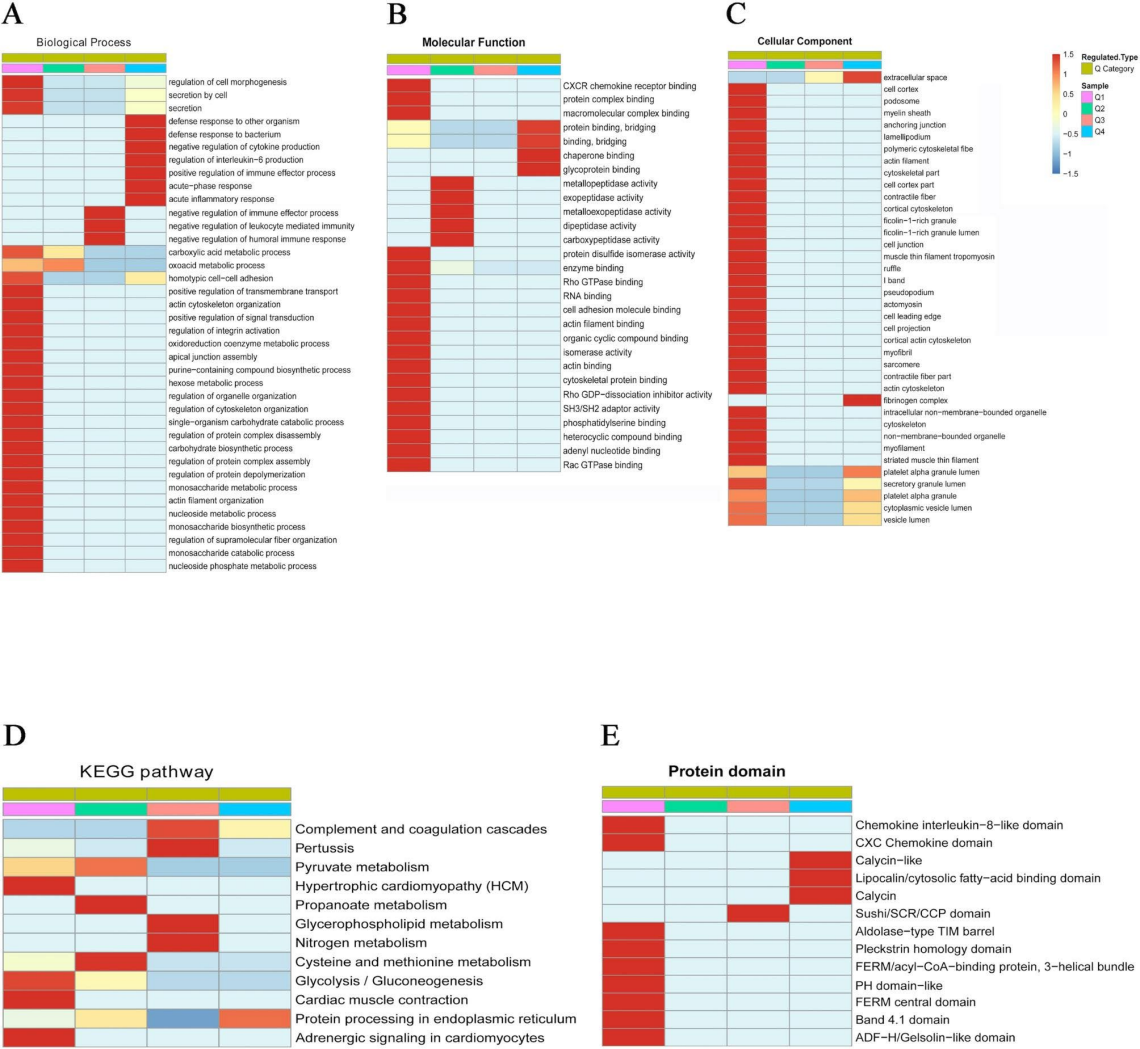
Cluster analysis was performed to determine the correlations between the functions of the differentially expressed proteins in the comparison groups. **A**: The cluster analysis of biological processes. **B**: The molecular function category analysis. **C**: The cellular component analysis. **D**: The KEGG pathway analysis. **E**: The protein domain analysis

In the cluster analysis for the biological process category, the upregulated proteins (Q3 and Q4) were highly enriched in the defense response, regulation of IL-6 production, acute-phase response, acute inflammatory response, immune effector process, negative regulation of cytokine production and leukocyte mediated immunity terms, all of which were associated with ORM1. In the downregulation group, the proteins were mainly enriched in secretion, secretion by cell and regulation of cell morphogenesis, positive regulation of transmembrane, regulation of integrin activation, while the secretion by cell and regulation of cell morphogenesis terms were associated with PPBP and SPARC (Fig. 2a). In the molecular function category, the proteins were highly enriched Q1 and Q2, including CXCR chemokine receptor binding, protein complex binding, macromolecular complex binding, which were associated with SPARC and PPBP. The upregulated proteins of cluster analysis, the proteins were enriched in the binding and protein binding terms, which were associated with vWF and ORM1, and in the chaperone binding term, which was associated with vWF (Fig. 2b). In the cellular component category, the enriched proteins were mostly downregulated proteins. Regarding immune function, only the extracellular space term was enriched, which was related to ORM1 among the upregulated proteins (Fig. 2c).

In the KEGG pathway cluster analysis, the proteins were mostly downregulated proteins; among the upregulated proteins, the proteins were enriched in complement and coagulation cascades, which were associated with vWF (Fig. 2d).

The domain category-based enrichment analysis indicated that the proteins were mostly enriched in downregulation groups. The chemokine interleukin-8-like domain and CXC chemokine domain were both associated with PPBP. Among the upregulated proteins, the calycin-like domain, lipocalin/cytosolic fatty-acid binding domain and calycin domain were all associated with ORM1 (Fig. 2e).

### Protein-protein interaction network

**Fig. 3:**
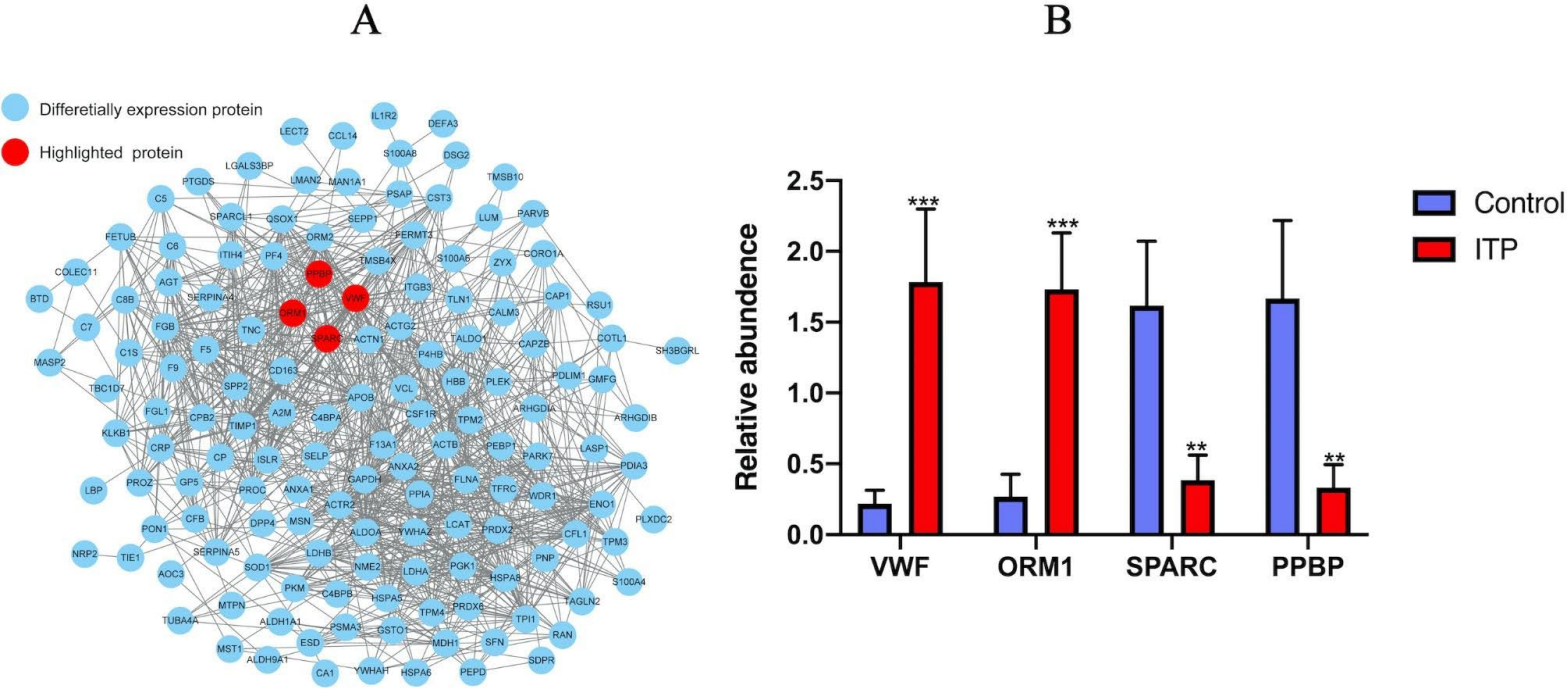
**A**: Protein-protein interactions of the differentially expressed proteins. The red bubbles represent the immune-related proteins ORM1, vWF, SPARC and PPBP; these four proteins interact with each other. The blue bubbles represent other differentially expressed proteins. **B**: Mass spectrometry-based targeted proteomics was conducted to quantify the four immune-related proteins. The levels of ORM1 and vWF were significantly higher in ITP samples than in control samples, while the levels of SPARC and PPBP were lower in ITP samples than in control samples. Two-tailed Student’s t-tests were used to compare the two groups. *P < 0.05, **P < 0.01, ***P < 0.001

To show the protein-protein interactions clearly, we selected the top 50 proteins with the closest interaction relationships and drew a protein-protein interaction network using the STRING (V.10.5) database. The immune-related proteins ORM1 and vWF were upregulated in the interaction network, while SPARC and PPBP were downregulated. The protein-protein network is depicted in Fig. 3a and the protein levels of ORM1 and vWF were clearly higher in ITP patients than in controls, while the protein levels of SPARC and PPBP were obviously lower in ITP patients than in controls (Fig. 3b).

## Discussion

ITP is an autoimmune disease, and loss of immune tolerance participates in its pathogenesis. In this study, we performed quantitative liquid chromatography tandem mass spectrometry (LC-MS/MS) analysis to identify differentially expressed proteins in ITP patients and normal controls. We found two upregulated proteins, ORM1 and vWF, and two downregulated proteins, PPBP and SPARC, associated with immune system processes by quantitative LC-MS/MS. We traced the four proteins according to proteins annotation, KEGG pathway and cluster analysis and found the four proteins were all related to the TNF-α signalling pathway, which of importance in the pathogenesis in ITP.

TNF-α is an important inflammatory cytokine whose levels are increased in some autoimmune diseases, such as SLE, rheumatoid arthritis, and inflammatory bowel disease [18–20]. Increased levels of TNF-α have also been found in ITP patient serum [21, 22]. Treatment using blockers of TNF-α has been found to increase the number of platelets in refractory ITP patients, indicating that TNF-α may be an important participant in the pathogenesis of ITP [23]. TNF-α binding to the TNF-α receptor induces a series of signal transduction pathways and thus regulates cell growth, differentiation, apoptosis and inflammation. Nuclear factor kappa B (NF-κB), mitogen-activated protein kinase (MAPK) and c-Jun N-terminal kinase (JNK) are important signalling pathways in the downstream regulatory pathway of TNF-α in the monocyte macrophage system [24].

In the biological processes enrichment of the differentially expressed proteins, the upregulated proteins were mainly enriched in the acute-phase response, acute inflammatory response, regulation of interleukin (IL)-6 production and regulation of leukocyte-mediated immunity terms, with which ORM1 was associated. ORM1 was found ORM1, also called α-1-acid glycoprotein (AGP1), is a glycoprotein with a low isoelectric point (p*I*) of 2.8–3.8 and a high carbohydrate content of 45% [25]. The biological function of ORM1 is related to its immunomodulatory properties and its ability to bind drugs [26, 27]. ORM1 is an acute-phase protein whose levels increase rapidly under conditions of inflammation, stress, chronic diseases (cancer, etc.) and injury and can be modulated by glucocorticoids, TNF-α, IL-1, IL-8, IL-6, and IL-6-related cytokines [28, 29]. Komori confirmed that ORM1 can concentration-dependently increase TNF-α, IL-6, and IL-10 levels above normal serum levels, plateauing at an acute-phase concentration, and found that phosphorylation of NF-κB, p38 and JNK occurs after incubation with ORM1 [30]. Higuchi confirmed that ORM1 can enhance NF-κB-mediated inflammation in vivo and found that ORM1 levels are higher in kidney transplant recipients pathologically diagnosed with chronic active antibody-mediated rejection than in kidney transplant recipients with normal histology [31]. These findings indicate the important role of ORM1 in immune disease. Therefore, in our study, the increase in ORM1 could have induced the expression of TNF-α directly or via activation of the NF-κB, p38 and JNK pathways, which are downstream of the TNF-α signalling pathway, to induce an immunological effect. vWF, one of the most common glycoproteins in the processes of thrombosis and haemostasis, is also a well-known index of endothelial damage and is involved in mediating the adhesion of platelets to subendothelial tissues [32]. In addition, vWF has been found to be involved in inflammation [33]. In the KEGG pathway analysis, vWF was found participates in the complement and coagulation cascades, which is well known. vWF was also found to participate in the ECM-receptor interaction, focal adhesion and PI3K-Akt signalling pathways, ultimately activating the NF-κB signalling pathway and MAPK signalling pathway; all of these pathways are associated with the TNF-α signalling pathway. Notably, Canobbio found that p38MAPK is phosphorylated upon platelet stimulation by vWF [34]. Bernado showed that proinflammatory TNF-α can stimulate the release of vWF from endothelial cells into the blood circulation [35].When TNF-α is increased, more vWF is released from endothelial cells. New findings have verified that vWF is associated with the inflammatory response in a direct or an indirect manner [33]. Nossent showed that the upregulation of vWF in SLE plays a role in immune-mediated inflammation, which is consistent with our hypothesis [36]. Similar to the case for ORM1, when vWF levels increased, the TNF-α signalling pathway was activated, thus inducing an immunological effect.

In the biological process cluster analysis, the regulation of cell morphogenesis and secretion by cell terms were associated with SPARC, a secreted protein that is acidic and rich in cysteines. SPARC is a small molecular glycoprotein secreted by endothelial cells and fibroblasts that inhibits cell adhesion, regulates the cell cycle and promotes tissue fibrosis. It is closely related to cell proliferation and angiogenesis [37]. SPARC has been found to modulate transforming growth factor (TGF)-β by phosphorylating Smad2 [38, 39]. TGF-β is a pleiotropic cytokine that regulates a broad range of cellular processes, such as differentiation, proliferation, migration, survival and apoptosis, and is downregulated in ITP patients [2, 40]. In the TGF-β signalling pathway, Smad2/3 can be suppressed by Smad6/7, which can be indirectly modulated by TNF-α [9]. However, SPARC deficiency in leukocytes results in exacerbation of inflammation owing to impaired TGF-β1-mediated TNF-α downregulation [41]. Taken together, these findings indicate that SPARC may be associated with cross-regulation of TNF-α and TGF-β in inflammatory infiltration. The downregulation of SPARC synthesis by inflammatory cytokines, including IL-1 and TNF-α, during the acute phase of arthritis, as verified by Nakamura, further supports our hypothesis about the role of SPARC in immune function and suggests that TNF-α can modulate SPARC reversal [42]. In our study, the downregulation of SPARC was consistent with the hypothesis that SPARC downregulation decreased TGF-β. In the cluster analysis of protein domains, the chemokine IL-8-like domain and the CXC chemokine domain were both associated with PPBP, which is also called CXC chemokine ligand 7 (CXCL7), and NAP-2, a platelet-derived growth factor belonging to the CXC chemokine family [43]. CXCL7 (PPBP) plays a prominent role in recruiting neutrophils to the injury site during thrombosis [44]. Dysregulation of CXCL7 has been implicated in inflammatory diseases, such as RA, acute lung injury and chronic obstructive pulmonary disease [45–47]. In addition, CXCL7 has been identified as a biomarker in lung cancer and advanced myelodysplastic syndrome [48, 49], suggesting that CXCL7 may participate in the progression of tumors. In the KEGG pathway analysis, PPBP was found to be participated in cytokine-cytokine receptor interactions and chemokine signalling pathways, which can indirectly modulate the MAPK signalling pathway. Wang showed that CXCL7 could be released by dendritic cells stimulated with TGF-β, although the function of TGF-β-induced CXCL7 was unknown. However, the findings support the view that CXCL7 may participate in the regulation of the immune response by inducing chronic inflammation or Th2 polarization [50]. In addition, Chiang found that CXCL7 expression can be inhibited by IL-6 [51], a downstream cytokine in the TNF-α signalling pathway, which is one of the mechanisms responsible for activating the host immune system against tumors. Therefore, we speculate that PPBP associates with the TNF-α signalling pathway via IL-6 and TNF-α downstream and plays an immunological role in Th2 polarization. In this study, PPBP was found to be downregulated; thus, it could weaken the polarization of Th2 cells, which is consistent with the findings of a previous study on Th1 polarization in ITP [52].

The analysis of protein-protein interactions indicated that the four proteins interacted with each other. According to the above points, we speculated that ORM1, SPARC, vWF and PPBP were involved in the TNF-α signalling pathway. ORM1 induced the NF-κB signalling pathway and induced the production of inflammatory cytokines, such as TNF-α and IL-6. vWF release was promoted by TNF-α, and vWF then participated in the PI3K-Akt signalling pathway, thus inducing the NF-κB and MAPK signalling pathways associated with the TNF-α signalling pathway. TNF-α reduced the production of TGF-β via Smad6/7, and TGF-β bound to SPARC to reduce TNF-α levels. Downregulation of SPARC weakened the reduction in TNF-α level in our study. CXCL7 (PPBP) could regulated TNF-α via IL-6, an important cytokine that is released downstream of the TNF-α signalling pathway, and IL-6 reduced the levels of CXCL7 (PPBP), while TGF-β induced CXCL7 expression. Similar to the case for SPARC, downregulation of PPBP weakened the polarization of Th2 and may have induced the polarization of Th1 cells. In summary, the four proteins are all immune-related and are all involved in the TNF-α signalling pathway in the pathogenesis of ITP.

## Conclusion

In general, this study provides a multilevel proteomic data resource for ITP research and increases understanding of the immunological function of ITP. The proteomic landscape of ITP is highly informative, identifying specific proteins. In fact, our comprehensive analysis revealed the key role of immunoregulation in the pathogenesis of ITP and the significant roles of the upregulated proteins ORM1 and vWF and the downregulated proteins SPARC and PPBP in the immunological function of ITP via LC-MS/MS. Our findings are of great significance for the diagnosis and treatment of ITP patients in the future. In the follow-up experiment, Western Blot, PCR, ELISA and vivo experiments would be conducted to verify the expression levels of ORM1, vWF, SPARC and PPBP. The function role of the four proteins would be detected by enhancing or blocking the proteins in TNF-α signalling pathway in ITP mouse models to find out the role of four proteins in the pathogenesis in ITP.

## Electronic supplementary material

Below is the link to the electronic supplementary material.

Supplementary Table S1: The KEGG-based functional enrichment analysis

Supplementary Fig. 1 A: The KEGG pathway of PI3K-Akt

Supplementary Fig. 1B: The TNF-α signalling pathway

Supplementary Material 4: The Full MaxQuant results

## Data Availability

All data generated or analysed during this study are included in this published article.

## Abbreviations

ITP: immune thrombocytopenia
ORM1: orosomucoid 1
vWF: von Willebrand factor
PPBP: pro-platelet basic protein
SPARC: secreted protein acidic and rich in cysteine
GPs: glycoproteins
MKs: megakaryocytes
IL: interleukin
LC-MS/MS: liquid chromatography tandem mass spectrometry
PRM: parallel reaction monitoring
TNF-α: tumor necrosis factor-α
TGF-β: transforming growth factor -β
NF-κB: nuclear factor kappa beta
MAPK: mitogen-activated protein kinase
JNK: c-Jun N-terminal kinase
TRAF6: tumor necrosis factor receptor-associated factor 6
SLE: systemic lupus erythematosus
RA: rheumatoid arthritis
IBD: inflammatory bowel disease
COPD: chronic obstructive pulmonary disease
CAAMR: chronic active antibody-mediated rejection
ALI: acute lung injury
GO: gene ontology
Q: quartile
AGP1: α-1-acid glycoprotein
CXCL7: CXC chemokine ligand 7
Th: T helper
