# Supplementary figures and images for: Identification of ORM1, vWF, SPARC, and PPBP as immune-related proteins involved in immune thrombocytopenia by quantitative LC-MS/MS
Source: Clin Proteomics. 2023 Jun 24;20:24. doi: 10.1186/s12014-023-09413-0 (PMC10290381; doi:10.1186/s12014-023-09413-0)

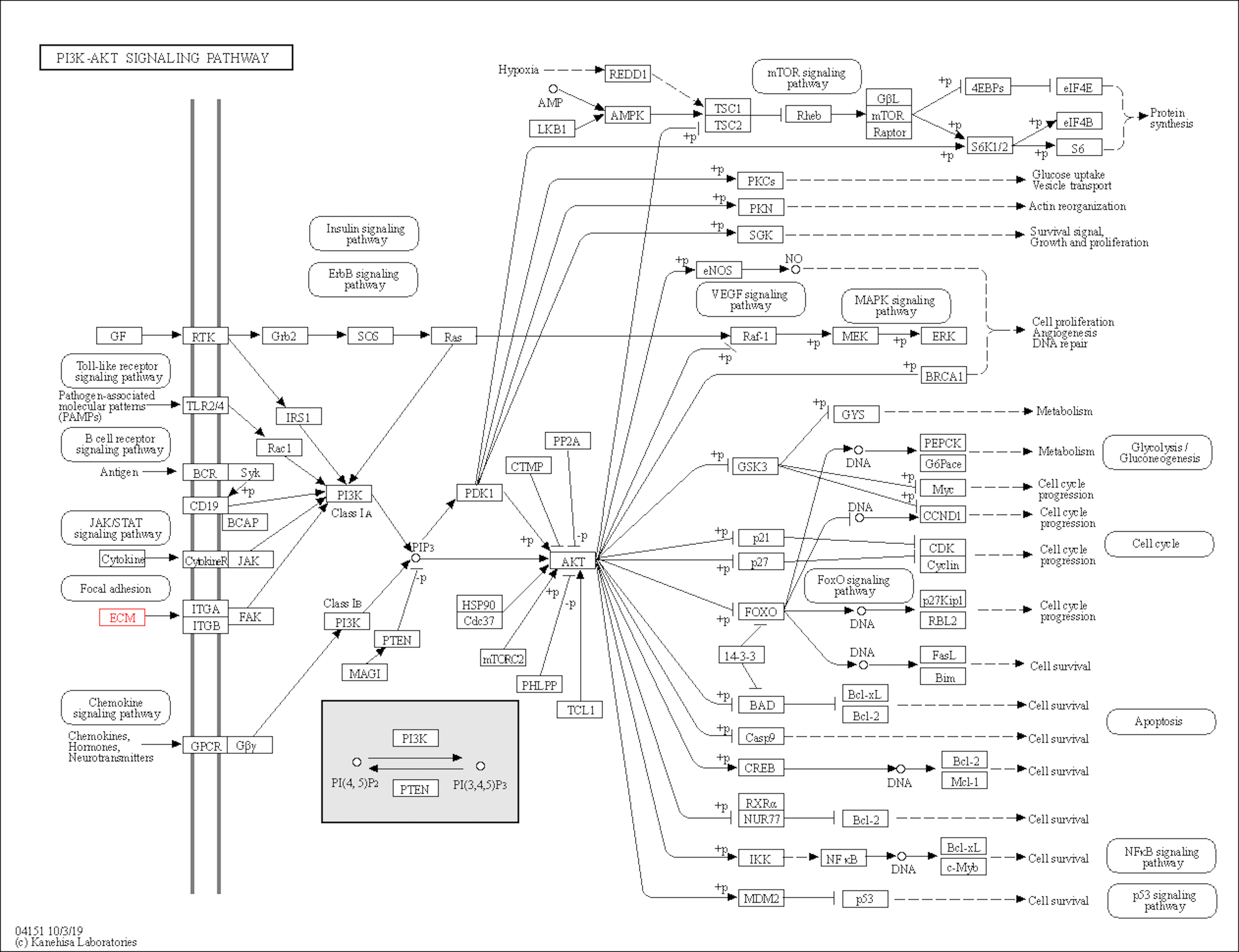

Supplement: Supplementary file 2 — Supplementary Fig. 1 A: The KEGG pathway of PI3K-Akt [file 12014_2023_9413_MOESM2_ESM.jpg]

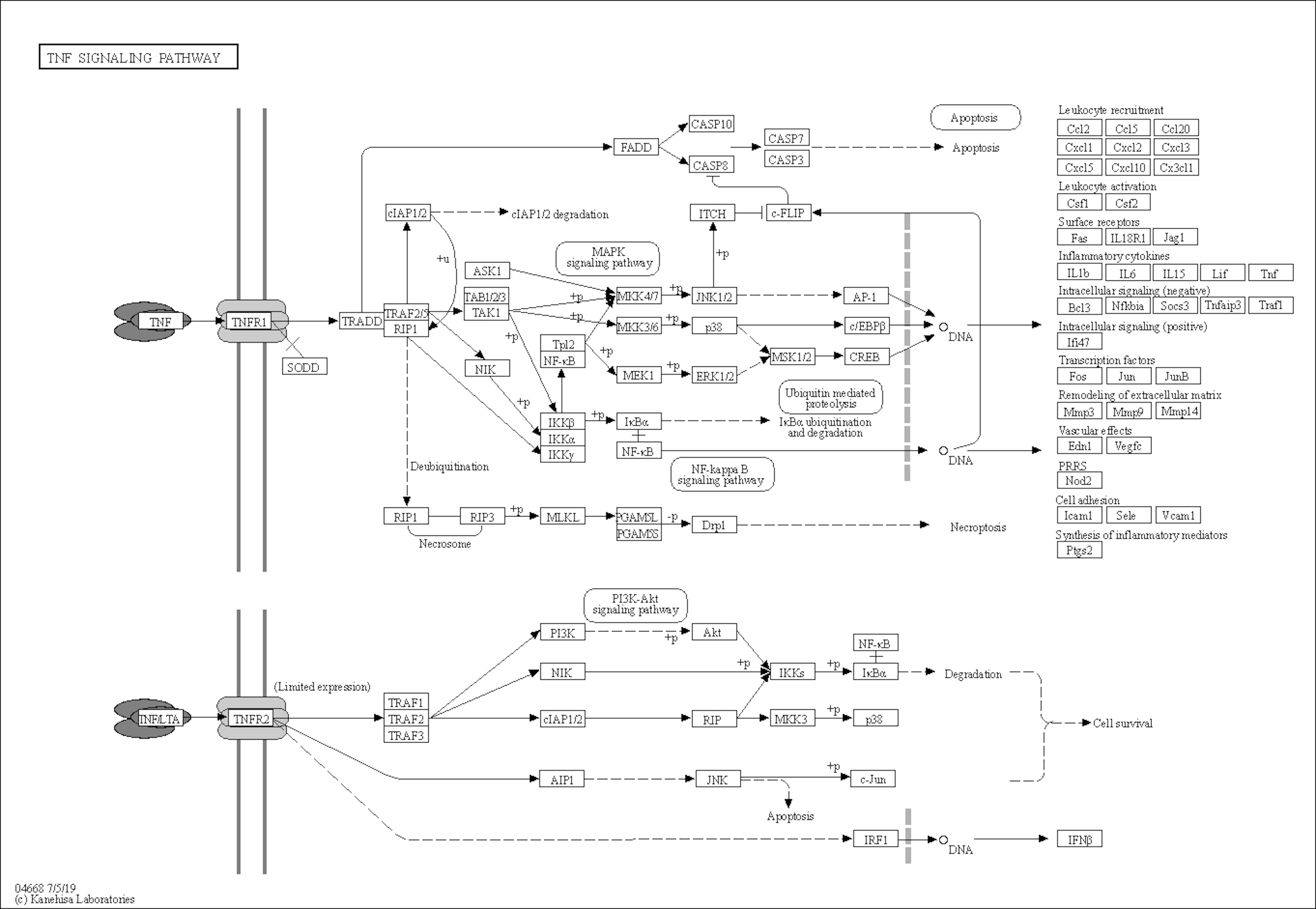

Supplement: Supplementary file 3 — Supplementary Fig. 1B: The TNF-α signalling pathway [file 12014_2023_9413_MOESM3_ESM.jpg]
